# Re-identification risk for common privacy preserving patient matching strategies when shared with de-identified demographics

**DOI:** 10.1093/jamia/ocaf183

**Published:** 2025-10-17

**Authors:** Austin Eliazar, James Thomas Brown, Sara Cinamon, Murat Kantarcioglu, Bradley Malin

**Affiliations:** HealthVerity Inc., Philadelphia, PA 19103, United States; HealthVerity Inc., Philadelphia, PA 19103, United States; HealthVerity Inc., Philadelphia, PA 19103, United States; Department of Computer Science, Virginia Tech, Blacksburg, VA 24061, United States; Department of Biomedical Informatics, School of Medicine, Vanderbilt University Medical Center, Nashville, TN 37235, United States; Department of Biostatistics, School of Medicine, Vanderbilt University Medical Center, Nashville, TN 37235, United States; Department of Computer Science, School of Engineering, Vanderbilt University, Nashville, TN 37235, United States

**Keywords:** privacy, record linkage, electronic medical records

## Abstract

**Objective:**

Privacy preserving record linkage (PPRL) refers to techniques used to identify which records refer to the same person across disparate datasets while safeguarding their identities. PPRL is increasingly relied upon to facilitate biomedical research. A common strategy encodes personally identifying information for comparison without disclosing underlying identifiers. As the scale of research datasets expands, it becomes crucial to reassess the privacy risks associated with these encodings. This paper highlights the potential re-identification risks of some of these encodings, demonstrating an attack that exploits encoding repetition across patients.

**Materials and Methods:**

The attack leverages repeated PPRL encoding values combined with common demographics shared during PPRL in the clear (e.g., 3-digit ZIP code) to distinguish encodings from one another and ultimately link them to identities in a reference dataset. Using US Census statistics and voter registries, we empirically estimate encodings’ re-identification risk against such an attack, while varying multiple factors that influence the risk.

**Results:**

Re-identification risk for PPRL encodings increases with population size, number of distinct encodings per patient, and amount of demographic information available. Commonly used encodings typically grow from <1% re-identification rate for datasets under one million individuals to 10%-20% for 250 million individuals.

**Discussion and Conclusion:**

Re-identification risk often remains low in smaller populations, but increases significantly at the larger scales increasingly encountered today. These risks are common in many PPRL implementations, although, as our work shows, they are avoidable. Choosing better tokens or matching tokens through a third party without the underlying demographics effectively eliminates these risks.

## Introduction

Many organizations in the United States seeking to link patient data are considered covered entities, or business associates, under the Health Insurance Portability and Accountability Act’s (HIPAA) Privacy Rule.^1^ While HIPAA does not apply to de-identified data,^1^ in the absence of a universal patient identifier, academic, government, and commercial entities frequently rely on privacy preserving record linkage (PPRL) techniques to ensure consistent patient identities across disparate datasets.^2^ The appeal of PPRL is its potential to support record linkage while ensuring that data remains de-identified, ensuring a more complete picture of the patient journey to support biomedical research and disease surveillance. It continues to be applied at increasing scale, including at the city,^3^ state,^4^ and national^5–9^ level. It is also increasingly accessible to implement with the development of open-source^4^^,^^10–12^ and commercial technologies.^2^^,^^13^

One of the main challenges in the implementation of PPRL is in the simultaneous optimization of (1) enabling accurate linkage of patient records across datasets while (2) minimizing a patient’s re-identification risk. To achieve these goals, it is standard practice for PPRL techniques to transform direct and quasi-identifying attributes, such as patient’s name, date of birth, and residential location, into encoded representations, often referred to as tokens, using cryptographic techniques. Encoding each attribute separately may make them vulnerable to frequency-based attacks, where some of the encoded values can be inferred based solely on how often they occur. Therefore, it is common to combine multiple attributes into a single token before encoding. Still, it is challenging to define a set of identifiers to be encoded that appropriately balance linkage precision and recall, while limiting the re-identification risk. The tradeoff depends on the specificity of the identifiers and whether one or more tokens (with different identifier sets) are applied in tandem.^2–4^^,^^10^^,^^13–18^

Prior literature has addressed the re-identification risks of auxiliary data in PPRL. These studies focus on quasi-identifying demographics, such as the age, race, marital status, and location of the individuals.^19–23^ As such, they evaluate the re-identification risk of the dataset instead of a risk posed by PPRL. These methods focus on the uniqueness of individuals based on these traits, therefore, the risk is reduced as the size of the underlying population grows. Although these vulnerabilities take advantage of the linking between records, only the demographics are considered in the risk assessment and they are independent of the linkage method or implementation used.

Previous research into the vulnerabilities of PPRL tokens have largely concentrated on attacks that utilize only the tokens themselves.^14^ Furthermore, these attacks have been demonstrated on datasets that are significantly smaller than those targeted by current large-scale PPRL implementations.^14^^,^^15^^,^^24^^,^^25^ With modern systems trending toward national representation,^5–9^ a token’s susceptibility to known attacks increases^15^ and tokens are exposed to new sources of re-identification risk. In this article, we examine one such emerging risk: the threat posed by the integration of PPRL tokens with other common demographics that may be shared during record linkage.

In this study, we evaluate the re-identification risk to PPRL tokens at scale by demonstrating a straightforward, yet effective, re-identification attack that exploits joint information between demographic information and repeated tokens. Unlike previous PPRL attacks, as the size of the dataset grows, so too does the feasibility and effectiveness of such attacks. We show that, while common tokenization strategies pose re-identification risks well under 1% of the population for datasets under one million individuals in size, the risk quickly grows and could lead to 10%-20% if implemented at the scale of national registries. Furthermore, we find that many other common PPRL strategies, such as the use of multiple tokens per patient (that is, tokens based on different combinations of a patient’s attributes), can exacerbate the risk, amplifying the privacy risks associated with large-scale PPRL deployments.

This study demonstrates a significant vulnerability in common PPRL designs. Notably, this risk is not inherent to PPRL itself and can be effectively mitigated through deliberate, informed implementation. By quantitatively assessing the risk and selecting appropriately designed tokens, the potential for exploitation can be rendered negligible. As our analysis demonstrates, many token formulations are not susceptible to this attack. Still, the adoption of well-regulated strategies, such as controlled centralized matching, can effectively avoid these risks by limiting or eliminating access to plaintext demographic attributes accompanying the tokens.

## Materials and methods

Various tokenization strategies are invoked in PPRL frameworks, often combining a patient’s name with demographic details, such as date of birth, to generate cryptographically encoded identifiers. Although these tokens are designed to be highly specific to an individual, often they are not truly unique. Common names lead to overlapping demographic traits, such as shared birth dates or locations, within large populations. This means that one token will often be shared by multiple individuals and additional demographic information is needed to differentiate them in the dataset. However, as we show in this work, the same demographic information can be leveraged by the data recipient to re-identify the underlying individuals.

We describe a practical re-identification attack that exploits repeated tokens and auxiliary demographic details. We assume, first, that the tokens are generated from publicly available information, such as described above. This is consistent with many tokenization strategies described in both academic and industry literature.^13^^,^^18^^,^^26^^,^^27^

Second, we assume that the tokens are shared alongside publicly available demographics, such as year of birth, gender, or the first 3 digits of a ZIP code (ZIP-3). These demographic values are frequently retained in de-identified datasets, including both HIPAA Safe Harbor practices and practices approved under expert determination. Notably, the attack presumes that the tokens and demographic information are available to the same party. If tokens are retained and processed separately from the de-identified records, such as by a third party performing the matching, the re-identification may not be feasible.

Third, we assume that the data recipient has access to a large reference database of identified individuals for comparison. Such reference datasets are a critical component in most re-identification attacks and may be obtained through publicly available records, telephone directories, voter rolls, or commercially aggregated data sources, as have been leveraged in prior re-identification attacks.^14^^,^^15^^,^^19^^,^^24^^,^^25^^,^^28^

### PPRL attack process

The attack consists of 4 basic steps: (1) grouping, (2) fingerprinting, (3) matching, and (4) identification. In the grouping step, we create a set of patient records that share the same token value. Each token group contains one or more patients.

Next, in the fingerprinting step, we create a distinctive descriptor for each token group. The descriptor corresponds to a list of each patient’s demographic information within the group. Demographics used within the token will stay the same for all patients in the group, but other demographics are expected to vary and create a list of values as long as the number of patients (with values allowed to repeat if there is more than patient with the same value). This provides a fingerprint of each group.

These first 2 steps are repeated independently for the tokenized dataset (the one that was matched with the PPRL system) and the reference data (which has the patients’ actual plaintext names and identities). The third step is matching groups across the 2 different datasets via the fingerprints. Specifically, if a fingerprint matches between 2 groups in the 2 datasets and is not shared by any other groups, we can confidently say that those groups are the same sets of patients. Therefore, we can unblind the token and match the encoded value to its corresponding plain text in the reference dataset.

Finally, in the identification step, patient records are linked to identified individuals using the unblinded token values and the demographics available in the de-identified and reference datasets. Occasionally, there will be patients who have identical quasi-identifying information within each group. These patients, though unblinded and possibly in a weakened privacy state, are not considered re-identified for the purpose of this paper.

Note that if the recipient has noisy or imperfect knowledge, Step 3 can be extended to include a constraint satisfaction problem (CSP) solver. For instance, if the reference data is a superset of the tokenized data (or vice versa), the matching is based on subsets of the fingerprint matching, and properties like mutual exclusion can be used to resolve many of the ambiguities and extend the matching results.

### An illustrative example

**Figure 1.**
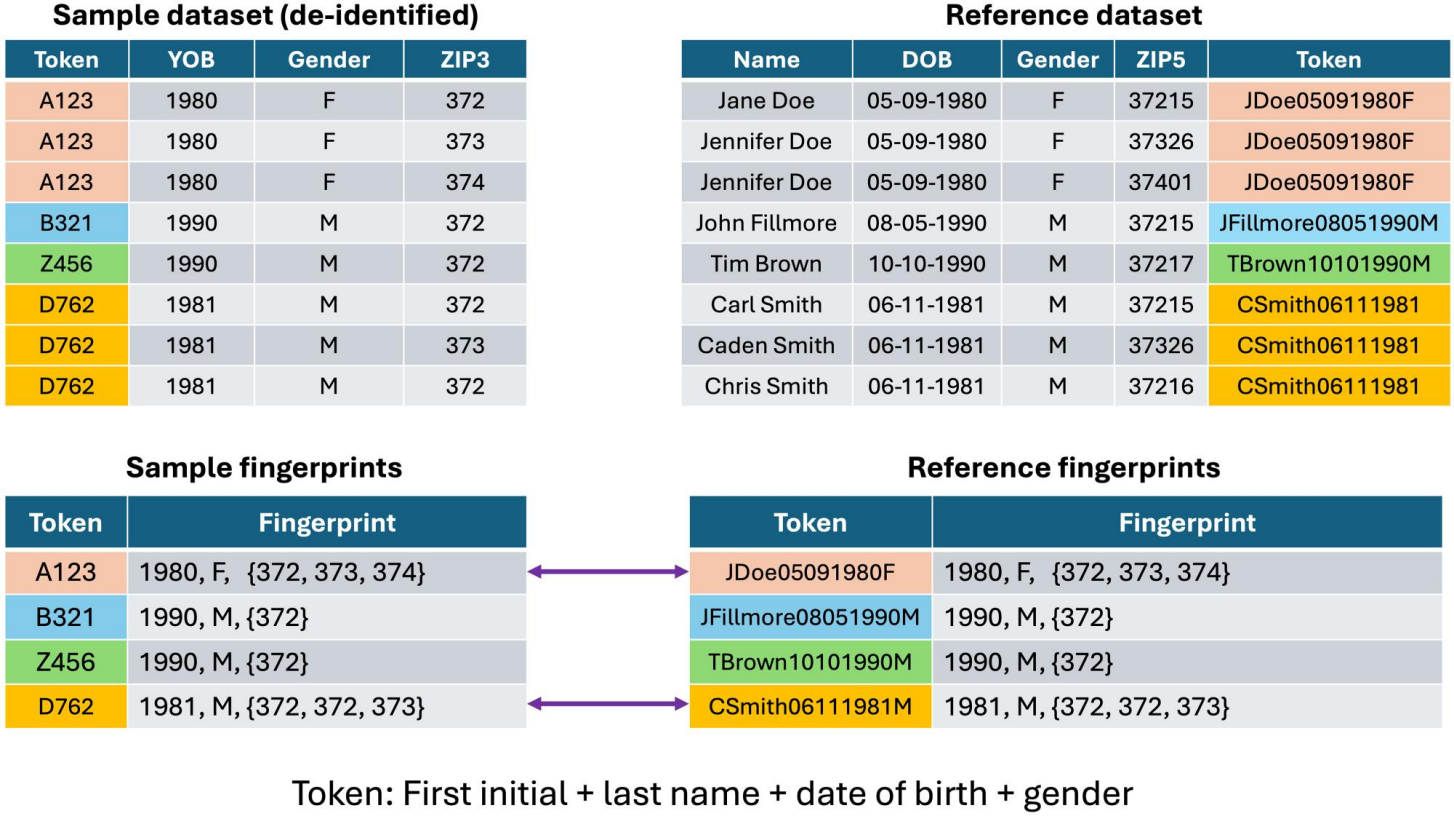
An example of the fingerprinting attack against a PPRL system.

To illustrate this attack, we consider a common token formulation that combines the patient’s first initial, last name, gender, and date of birth, as shown in Figure 1. For instance, a token Jennifer Doe is represented as {jdoe05-09-1990F}. This string is hashed into a secure, non-reversible token.

Each individual in the dataset is assigned a token derived from the (hidden) patient personally identifying information. That token is joined to the patient’s record in the dataset, which includes common demographic fields.

The attack begins by extracting a list of all unique tokens, as shown at the top of the figure. In our example, the first 3 people in the list share the same token, as do the last 3, as shown by the different colors. These individuals with shared tokens are grouped together, and each token is fingerprinted with the shared year of birth and the set of ZIP codes associated with that token (bottom of Figure 1).

The next step involves acquiring a reference database of identifiable individuals containing the same components in plaintext format for comparison purposes, as shown on the right side of the figure. The recipient generates corresponding plaintext tokens from this reference and fingerprints them using year of birth, gender, and ZIP3 values, mirroring the target dataset.

Comparing the hashed tokens from the de-identified dataset on the left with the plaintext tokens in the reference on the right, the fingerprints can be seen to match. For instance, the token A123 is clearly associated with the individuals *J. Doe*, born on May 9, 1990. In many cases, particularly when a token is linked to several ZIP-3 values, only one reference entry will match, enabling a high-confidence re-identification.

### Attack process for multiple tokens

One PPRL strategy that is commonly employed is to use multiple tokens with different encoding methods for each patient.^3^^,^^4^^,^^13^^,^^17^^,^^18^ This greater redundancy from an ensemble of tokens provides improved resilience to noise and ambiguity in the data to increase the precision and recall of the linkage. Unfortunately, the added tokens also provide greater risk to re-identification.^15^ We describe here a simple extension for the proposed attack that includes the additional information of 2 tokens. This extension is notional and more sophisticated attacks are possible. A complete treatment of the problem for multiple tokens is outside the scope of this article.

Assume that each individual in the dataset has 2 distinct tokens, X and Y, based on different combinations of the patient demographics. Using the same attack algorithm as described earlier, 2 tokens give each individual 2 fingerprints. A naïve extension of the algorithm would use each fingerprint independently. Either fingerprint might be unique for that token, increasing the chance of re-identification.

However, using 2 tokens also means that the risk can propagate. Once an individual is re-identified based on one token, that unblinds the second token, allowing more individuals to be re-identified. We consider an extended attack consisting of 6 basic steps: (1) token grouping, (2) token fingerprinting, (3) individual fingerprinting, (4) matching, (5) identification, (6) propagation.

The first 2 steps of token grouping and fingerprinting are performed the same as above, but for both token X and token Y. The result is that each token group is characterized by a fingerprint of quasi-identifiers. Step 3 extends the fingerprinting to specific individuals, where an individual person is characterized by the concatenation of the fingerprint from their value for token X, the fingerprint from their value for token Y, and the demographics for that person. In certain cases, neither the fingerprint for token X nor token Y are sufficiently unique to identify an individual. However, the combination of the 2 may be unique for a specific individual. This allows additional individuals to be re-identified that were not vulnerable under either token by itself.

Matching (Step 4) is similar to the matching step of the base attack. If a fingerprint matches between 2 groups of the same type—a group from token X, from token Y, or an individual—and is not shared by any others of the same type, those represent the same group or individual.

Identification (Step 5) is the same as the identification step of the base attack.

Propagation (Step 6) propagates information revealed by the identification step. Once a specific individual is identified, all of the tokens associated with that individual are unblinded, and the corresponding token groups can be matched. These new matchings are used to revisit Step 5 and potentially identify a new set of individuals. Identification and propagation iterate until no new matchings are revealed.

### Experimental design

We investigate 6 tokens that have been reported in the literature to appraise the efficacy of the attack, based on prevalence of use and diversity of the patient-specific attributes.^3^^,^^4^^,^^10^^,^^13^ These tokens are:

- Soundex(first name) + Soundex(last name) + date of birth + gender
- First letter(first name) + last name + date of birth + gender
- Soundex(first name) + Soundex(last name) + ZIP3
- First name + last name + date of birth + ZIP code
- Soundex(first name) + Soundex(last name) + date of birth
- First letter(first name) + last name + date of birth

Soundex is a phonetic algorithm that encodes names based on their pronunciation in English, enabling the matching of names with minor spelling variations.

Our experiments are based on data derived from the voter registration lists of Florida, North Carolina, and Ohio. We synthesized one element for the North Carolina data. This is because this resource records the year of birth of the voter as opposed to the date of birth. For these records, a date of birth was generated by randomly sampling from the distribution of dates in Ohio and Florida for the given year of birth. Individuals over 90 years old were excluded, while the voter registry itself only included individuals at least 18 years old. Any individuals with missing or suppressed information were removed. This provided a reference set of 30 927 286 individuals with ages ranging from 18 to 90 from 74 distinct 3-digit zip codes.

In our experimental results, we excluded individuals who could be uniquely identified based on demographic combinations alone (e.g., a unique year of birth, gender, and ZIP3). These individuals could be re-identified using known methods with or without the tokens. The reported re-identification rates reflect only the *incremental* risk introduced by the tokenization strategy, isolating the additional risk presented by including the token with the records.

## Results

### Unblinding and re-identification

To establish the baseline privacy risk for each token, in terms of unblinding and re-identification, we performed random sampling of 25 million individuals without replacement from all 3 states. Each sample dataset was encoded using the chosen tokenization strategy, along with observable attributes: gender, year of birth, and ZIP3. Table 1 reports the percentage of the population successfully unblinded (identifying the plaintext for the token) vs. the population that uniquely re-identified to a single specific person. While the risks vary dramatically between the different tokenization strategies, each tokenization strategy consistently shows that the difference between unblinded and re-identified was minimal—the demographic quasi-identifiers were sufficient to disambiguate between the individuals within a specific token for the vast majority of cases. Given the similarity between these measures, we focus solely on re-identification for the rest of the experiments.

**Table 1.** The unblinding and re-identification rates for the 25 million patient sample based on FL, NC, and OH voter registration lists.

	Tokens					
	A	B	C	D	E	F
**Unblinding percentage (SD)**	0.59 (0.0012)	0.94 (0.0026)	48.99 (0.0042)	0.00 (-)	0.83 (0.0022)	1.99 (0.0028)
**Re-identification percentage (SD)**	0.57 (0.0011)	0.93 (0.0026)	45.23 (0.0041)	0.00 (-)	0.81 (0.0022)	1.94 (0.0028)

### Risk at state scale

Our first set of experiments examined how dataset size impacts re-identification risk by varying the sampled population size. For each combination of token and population size, we conducted 20 independent trials and report the average results. This was repeated for each state and a combined dataset of all 3 states. The SD across trials was less than 0.008% for all experiments, which is too small to plot on the figures.

**Figure 2.**
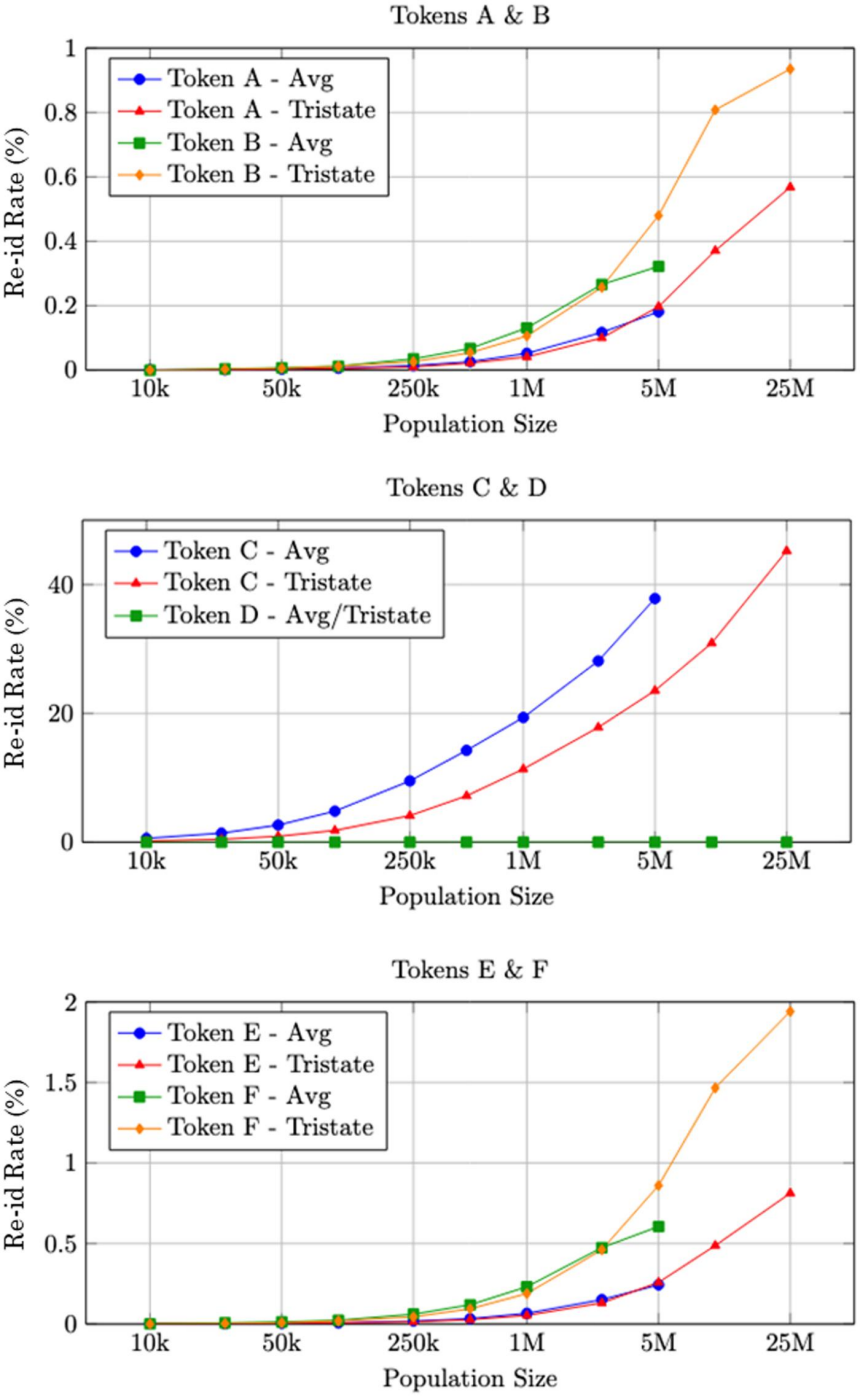
The re-identification rate of different tokenization strategies on state-level populations. The Avg results depict the average re-identification risk across each of the 3 states separately. The Tristate results dept the results from combining all 3 states into a single dataset. Note that the *y*-axis has a very different scale for each of the 3 pairs of tokens.

Figure 2 illustrates the results, which show that re-identification risk is strongly associated with both token structure and dataset size. Most tokens exhibited rates below 1% for population sizes up to 5 million, but increased sharply as populations approached 25 million. This highlights a consistent trend of significant re-identification risk as we interact with larger datasets, suggesting a growing problem at national scales.

Comparative analysis of token structures revealed that even minor differences in a token’s attributes can significantly affect re-identification. For example, Tokens A and E differ only in the inclusion of gender, but there is a 1.4× increase in risk. A similar pattern is seen in Tokens B and F.

Token C, which substitutes ZIP3 for date of birth, consistently produced the highest re-identification rates, necessitating separate visualization due to scale. The coarseness of ZIP3 leads to many individuals sharing the same token, significantly reducing uniqueness and increasing vulnerability.

By contrast, Token D, which combines full names with date of birth and full ZIP code, exhibited the lowest re-identification rates across all population sizes. This token’s precision resulted in near-unique tokens, thereby preserving privacy even at large scales.

The key finding of Token D is that not all tokenization strategies are vulnerable to this attack. Numerous token encodings exist that are sufficiently unique to one specific individual. This uniqueness renders these records impervious to all of the attacks of this type. This observation underscores that the risk is not intrinsic to PPRL as a whole, but rather arises from specific implementation choices; most notably, the design of the token encodings themselves.

These findings underscore that not all tokens are equal in their risk. Small variations in the attributes used for tokenization can have profound implications for re-identification vulnerability and the selection of what information to include in a tokenization strategy is critical to the privacy risk.

### Risk at national scale

To investigate re-identification risk at a national scale, we used a Monte Carlo simulation based on the Florida, North Carolina, and Ohio voter registration lists modified to reflect a representative U.S. population. Specifically, we simulated larger populations by sampling combinations of name, gender, and year of birth with replacement from the original dataset. Full date of birth was imputed based on the empirical distribution within each birth year. To expand geographic diversity, ZIP3s were drawn from all U.S. ZIP3 codes with a population of at least 20 000 (per the 2020 Census), sampled proportionally to their populations.

This method generated synthetic datasets with realistic demographic and geographic characteristics representing hundreds of millions of individuals. As the simulated population increased, re-identification rates rose sharply, aligning with earlier trends and underscoring the rapid increase in privacy risk when token-based linkage is applied at national scales.

The Monte Carlo simulation employed in this study relies on several assumptions of independence within the underlying data. See the Appendix S1 for a sensitivity analysis of these independence assumptions.

### Attacks that combine multiple tokens per patient

The previous experiments focused on the risk of a single token at a time; however, PPRL systems commonly exchange multiple tokens per individual to account for errors and noise.^3^^,^^4^^,^^13^^,^^17^^,^^18^ While this technique aims to improve linkage accuracy, it also increases the potential for attack. To assess this additional risk, we performed an experiment using Tokens A and B simultaneously. These tokens differ slightly in their structure but share several underlying attributes. The method for combining the token is described in Attack Process for Multiple Tokens section, above.

As the results in Figure 4 illustrate, when both tokens are shared to the recipient, the re-identification rates increased substantially. Thus, the use of token ensembles, though common, can exacerbate re-identification vulnerabilities in unexpected ways and should be employed with caution.

### Attacks with imperfect knowledge

In this experiment, we consider the scenario where the recipient has access to a reference plaintext dataset that is a superset of the tokenized population. In this attack, a token is matched to the plaintext if the token’s fingerprint is the only one that is the same or a subset of the plaintext version. In this case, the recipient can benefit from principles of mutual exclusion to help refine the set of candidate matches.

We tested the results on a simulation of a nationally representative population. The experimental results in Table 2 show that this while imperfect knowledge can mitigate the risk somewhat, it is entirely ineffective at small populations and only has limited effect at larger populations. For a population of 50 million adult patients, a recipient with a reference set of 200 million individuals—nearly the full US adult population—can achieve re-identification rates of 66% of those that could be achieved with perfect knowledge.

**Table 2.** Effect of imperfect knowledge on re-identification risk on Token B.

Tokenized population	Reference data size (vs. tokenized population)			
	1×	2×	3×	4×
100 000	0.0%	0.0%	0.0%	0.0%
250 000	0.0%	0.0%	0.0%	0.0%
500 000	0.1%	0.1%	0.1%	0.1%
1 000 000	0.1%	0.1%	0.1%	0.1%
2 500 000	0.3%	0.3%	0.3%	0.3%
5 000 000	0.5%	0.5%	0.5%	0.5%
10 000 000	1.0%	1.0%	1.0%	1.0%
25 000 000	2.4%	2.3%	2.2%	2.1%
50 000 000	4.4%	3.9%	3.4%	2.9%

### Rate of non-unique tokens

Next, we assessed the theoretical upper bound of re-identification risk by measuring the proportion of individuals who share their token with at least one other person, i.e., those with non-unique tokens. Since the proposed attack depends on overlapping token values, individuals with unique tokens are inherently protected under this threat model.

We calculated the percentage of non-unique tokens across a range of population sizes for various token types, the results of which are shown in Table 3. These numbers provide a conservative estimate of the maximum re-identification rate, assuming the recipient has perfect auxiliary knowledge and sufficient fingerprinting power to resolve conflicts. The results show that the non-unique token rate increases approximately linearly with population size, reinforcing our observation that larger datasets inherently pose greater privacy risks under this attack framework.

**Table 3.** The rate of individuals who have a non-unique token across varying population sizes.

Population size	Percent with a non-unique Token			
	Token A	Token B	Token C	Token D
10 000	0.000%	0.001%	0.194%	0.000%
20 000	0.001%	0.003%	0.403%	0.000%
40 000	0.002%	0.005%	0.796%	0.000%
80 000	0.004%	0.008%	1.506%	0.000%
160 000	0.006%	0.016%	2.896%	0.000%
320 000	0.014%	0.033%	5.260%	0.000%
640 000	0.026%	0.069%	9.261%	0.000%
1 280 000	0.052%	0.135%	15.341%	0.000%
2 560 000	0.104%	0.268%	23.762%	0.001%
5 120 000	0.208%	0.529%	34.258%	0.001%
10 240 000	0.414%	1.036%	46.040%	0.002%
20 480 000	0.820%	1.994%	57.969%	0.005%

### ZIP3 fingerprint

ZIP3 diversity significantly influences token fingerprinting.

To examine this effect, we restricted the tokenized population to smaller geographic areas with fewer ZIP3s. Starting with a random selection of 30 ZIP3s, we measured re-identification rates for Token A and Token B across varying sample sizes. We then gradually increased the number of ZIP3s while repeating the analysis.

**Figure 3.**
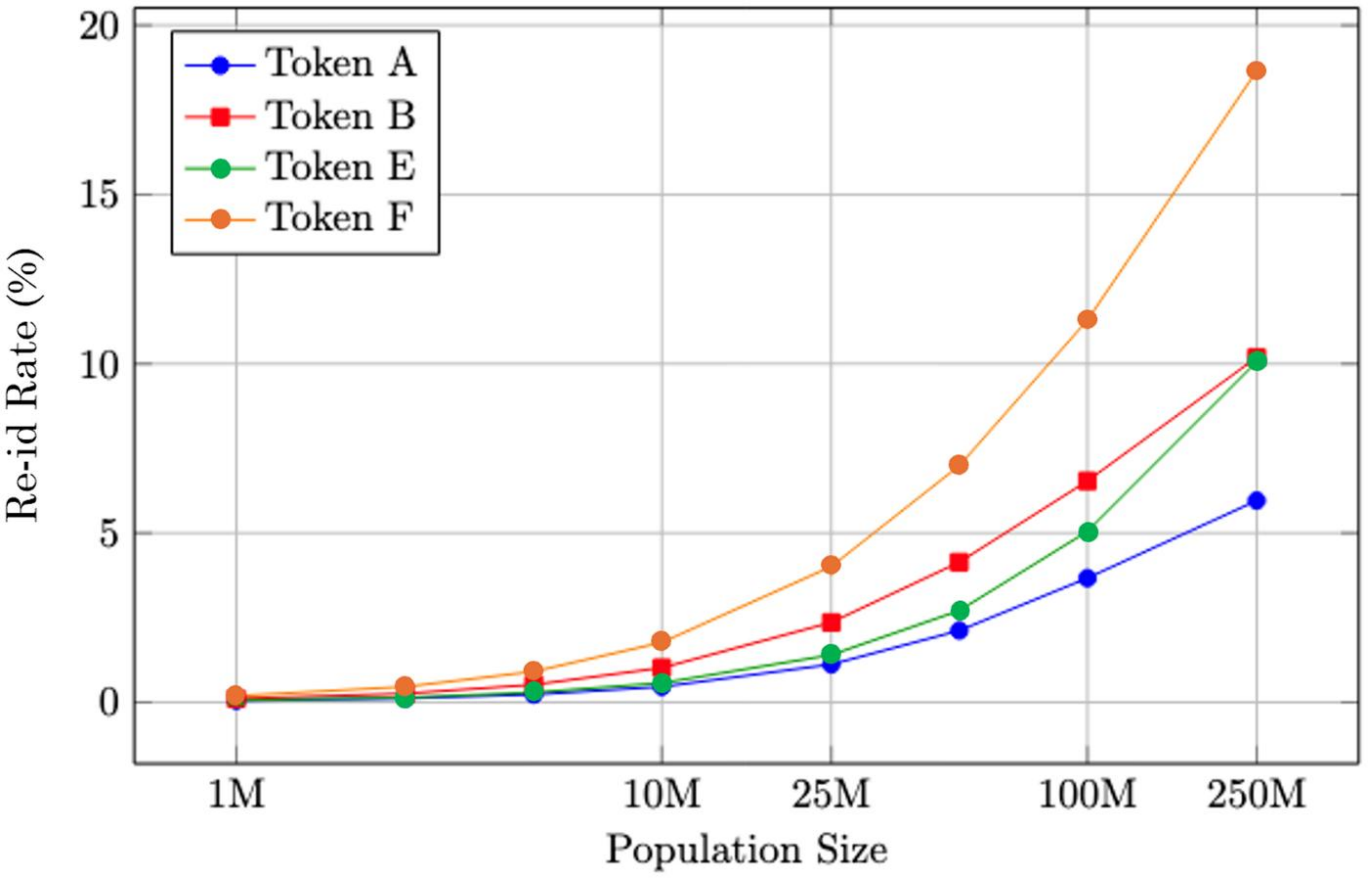
Risk of different tokenization strategies on national scales of population and ZIP codes.

As shown in Figure 5, re-identification rates initially rise with population size but eventually plateau within constrained ZIP3 regions. This is due to token fingerprint saturation—new tokens increasingly share the same ZIP3 values, reducing uniqueness. In contrast, broader geographic datasets (e.g., one covering over 900 ZIP3s nationally) maintain higher fingerprint diversity, allowing re-identification risk to continue rising with scale. This saturation effect helps explain why we continue to see risk rise in nationally representative population sizes in Figure 3.

**Figure 4.**
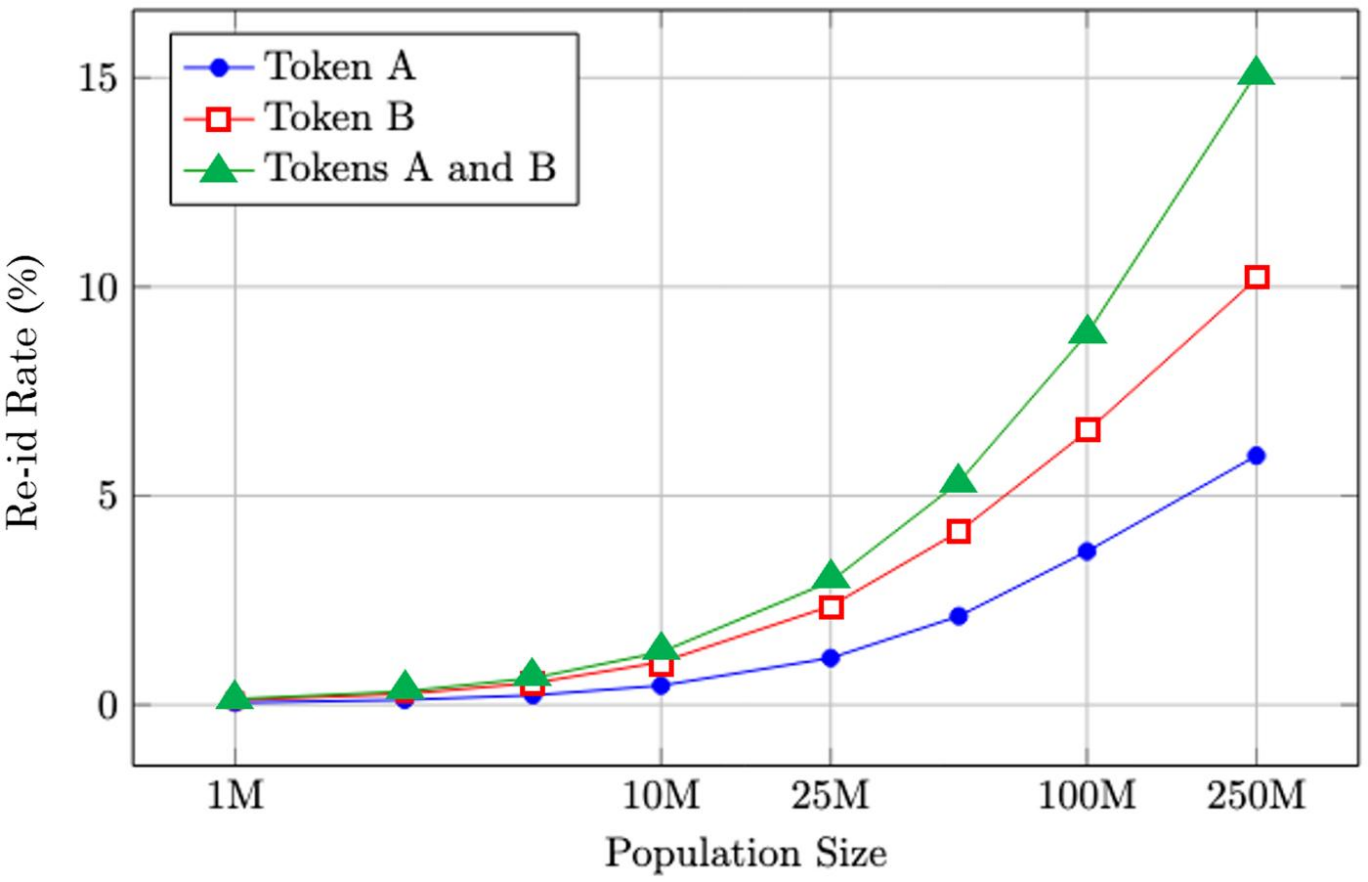
Re-identification risk when combining Tokens A and B.

**Figure 5.**
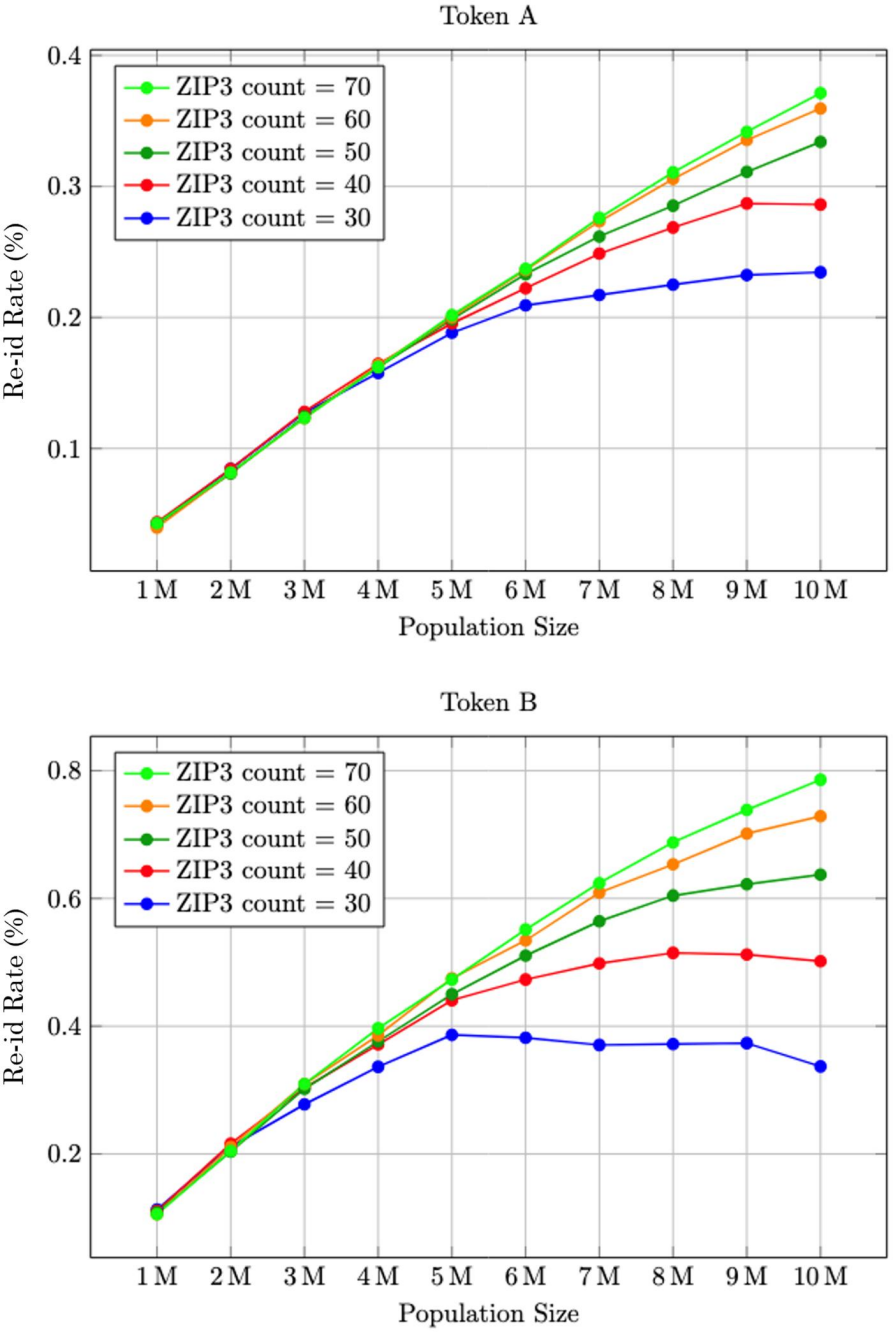
Effect on re-identification risk from varying the granularity of ZIP-3 for Token A (top) and Token B (bottom). ZIP-3 is not included in the creation of either token, so the effects are limited.

### Year of birth fingerprint

**Figure 6.**
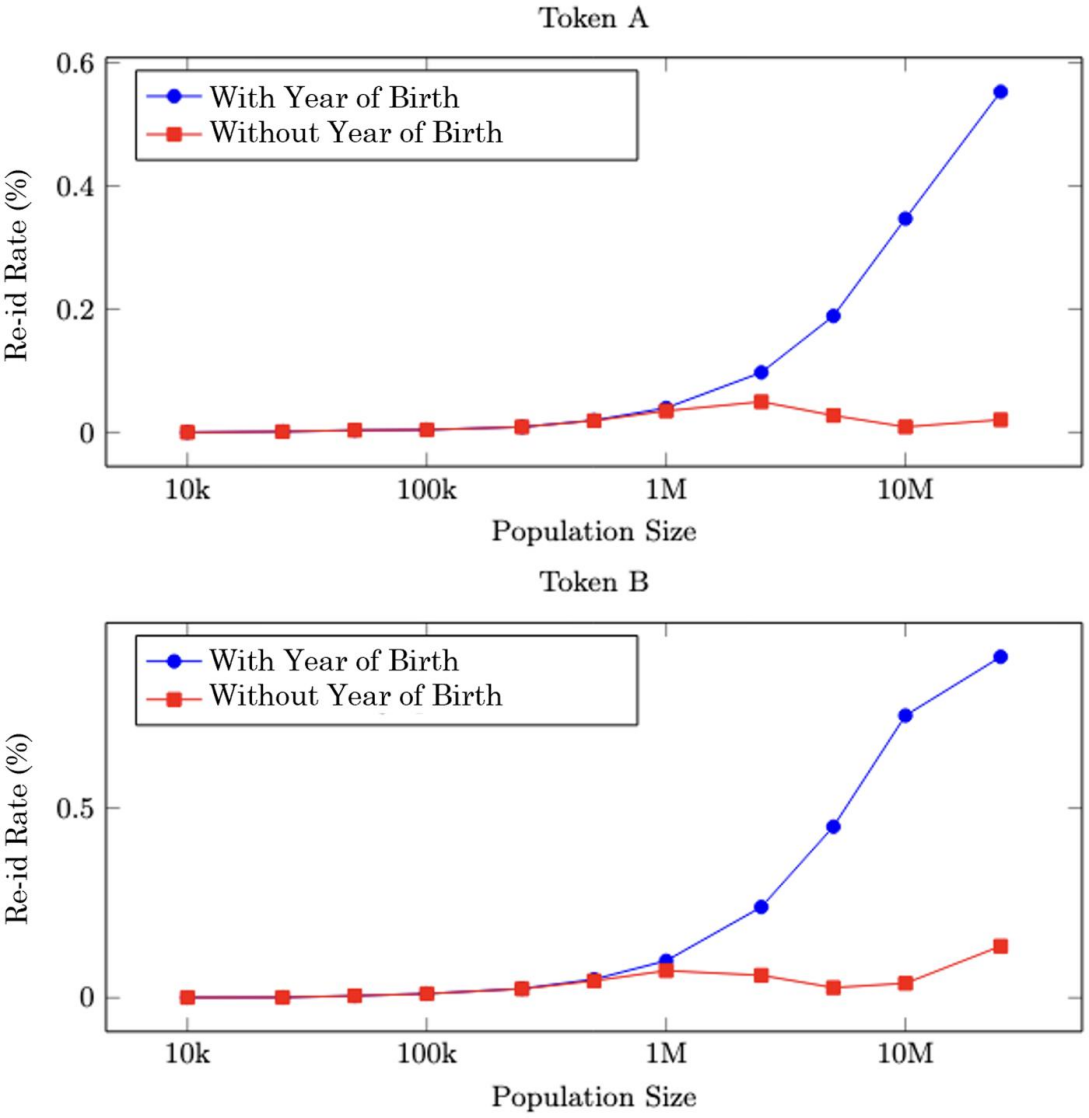
Effect on the re-identification risk of removing the year of birth from the dataset for Token A (top) and Token B (bottom). The year of birth is part of the formulation for both tokens, so the effects of removing it from the dataset are more severe than removing an unrelated demographic, such as shown in Figure 5.

The final experiment evaluated the sensitivity of the re-identification attack to the availability of demographic information used in creating the tokens. Specifically, using both Token A and Token B across datasets from 3 US states, we compared re-identification rates under 2 conditions: with and without the inclusion of patients’ year of birth, as shown in Figure 6. Although the year of birth is embedded within the token in both tokenization strategies—thus appearing only once per fingerprint—it is important in reducing the pool of available candidates that could match to a specific token from the reference dataset. Therefore, its availability in the dataset had a pronounced impact on re-identification outcomes. The attack demonstrated only minimal effectiveness when the year of birth could not be included in the fingerprint, highlighting its critical role in linking records.

These findings point to a potential risk mitigation strategy based on excluding the year of birth. However, they also underscore the broader risk that may arise from the inclusion of additional demographic attributes. In particular, latent demographic signals inferred from healthcare data—such as race, ethnicity, socioeconomic status, or marital status—may similarly elevate privacy risk when used in conjunction with PPRL tokens.

## Discussion

This study shows that large-scale PPRL systems can pose significant re-identification risks when tokens are shared alongside quasi-identifiers. While the risk is minimal in datasets with fewer than one million individuals, it increases rapidly with scale. In populations representative of the United States, over 15%-20% of individuals could be re-identified using commonly used tokens. Alternative tokenization schemes—such as employing multiple tokens or using Token C—rise to over 45%, even in smaller datasets.

We highlight that these estimates are conservative for some real-world systems. Systems that include more tokens, tokens with more repeated values, and/or additional quasi-identifiers (e.g., race, ethnicity, marital status, education level, etc.) will increase the risk beyond this study’s estimates. For such systems, the estimates should be considered lower bounds.

Nonetheless, the findings also suggest practical mitigation strategies. Tokens are not inherently vulnerable; the risk arises from their combination with publicly available demographic information. One of the most effective defenses is to avoid exposing both tokens and plaintext demographics together. This can be achieved by separating tokens from other identifying attributes during processing.

A centralized matching architecture offers one solution: tokens are sent without accompanying demographics to a third party that performs the linkage and returns identities. Fingerprinting risk can be further reduced by limiting, encoding, or removing demographic variables, and restricting access to sensitive data. To ensure returned identities are not similarly vulnerable, each should be limited to a single value per demographic category.

Lastly, token design should account for system scale. Adjusting token specificity and minimizing the number of tokens used can help control risk while maintaining matching accuracy. However, as systems grow, tokens may need to be recalibrated to maintain acceptable risk levels. Overall, scalable PPRL is feasible, but it requires deliberate design choices and strong safeguards to minimize re-identification risks.

We highlight several limitations to guide future extensions. First, the records for the experiments were based on a subpopulation of 31 million individuals aged 18-90 who had registered to vote. Although this represents 82% of the adult population in those 3 states, the remaining population may contain different distributions of names or zip codes and latent correlations that could bias the risk estimates when extrapolating to larger populations. For healthcare datasets that include minors, risk estimates may be biased if the child population has significantly different distributions than the adults. Future work should account for potential representation biases.

Second, the risk assessments presented assume the healthcare datasets are an unbiased sampling of the national population, including latent populations. Real world datasets are typically a biased selection of individuals, either explicitly due to the specific population and selection criteria under analysis, or implicitly due to sourcing limitations from data providers and the healthcare institutions that they work with. For instance, a national Medicare-based dataset may have a very different risk profile than a New England-based pediatric cancer registry. Understanding the effects of these significant population variations is important to understanding the risk potential for real-world use cases and should be considered in future work.

## Conclusions

In this article, we identified a potential privacy risk for PPRL systems when sharing cryptographically hashed tokens combined with quasi-identifiers. We detailed a practical attack capable of exploiting the vulnerability and re-identifying a major portion of the individuals in a de-identified dataset. While most populations under 5 million individuals have a re-identification rate under 1%, the rate grows quickly with the size of the population and can exceed 10%-20% at nationally representative populations. Certain tokens or combinations of tokens showed much higher rates even at lower populations, while other tokens had negligible risk, highlighting the need for careful consideration of the strategy and the scenario when assessing risk.

We presented several options for mitigation of this risk. First, evaluation and selection of more secure tokens can effectively negate the vulnerability. Second, centralized matching by a trusted third party can decouple the tokens from sensitive attributes. Finally, removal of plain-text demographics from the dataset can negate fingerprinting elements for the attack. Ideally, multiple options can be used concurrently to minimize the risk.

## Supplementary Material

ocaf183_Supplementary_Data

## Data Availability

The Florida voter data underlying this article are available from the Florida Board of Elections by request: https://dos.fl.gov/elections/data-statistics/voter-registration-statistics/voter-extract-request The North Carolina voter data is available from the North Carolina Board of Elections at: https://www.ncsbe.gov/results-data/voter-history-data The Ohio voter data is available from the Ohio Secretary of State at: https://www6.ohiosos.gov/ords/f
