# Supplementary material for: Re-identification risk for common privacy preserving patient matching strategies when shared with de-identified demographics
Source: J Am Med Inform Assoc. 2025 Oct 17;33(2):336–46. doi: 10.1093/jamia/ocaf183 (PMC12844594; doi:10.1093/jamia/ocaf183)
Supplement: ocaf183_Supplementary_Data [file ocaf183_supplementary_data.docx]

# Sensitivity Analysis of Monte Carlo Simulation

First, we assumed demographic variables occurred independently when estimating national re-identification risk from Monte Carlo simulations. This assumption does not account for real-world correlations – such as surnames occurring more frequently in certain geographies or racial groups – that would likely increase the occurrence and distinguishability of repeated token values. This provides another reason our risk estimates should be considered as lower bounds. Future work should account for such correlations.

When estimating national re-identification risk from Monte Carlo simulation, we assumed certain demographic variables occurred randomly, while others were correlated. Age, gender, and 3-digit ZIP code were sampled based on joint distributions from the 2020 U.S. Census. The name was sampled according to age and gender based on conditional distributions from the three state voter registries. When age was outside of our voter registries’ range, names were sampled independent of age. The birthday was similarly sampled according to age, but independent of name. These independence assumptions do not account for real-world correlations – such as surnames occurring more frequently in certain geographies – that could affect the occurrence and distinguishability of repeated token values.

We tested the sensitivity of risk for these independence assumptions by comparing known data to simulated data under these assumptions. For each of the three states, we generated a simulated data set of the same size population, using the same Monte Carlo sampling strategy. When sampling from the national Census data, we restricted the Census data to that state only. Ages were restricted to a range of 18-90 to be consistent with the slice of the population represented by the voter registries.

We tested the re-identification rate of each token on each simulated state, and the combined set of all three states. Sampling was repeated ten times each, with the re-identification rate averaged across the ten simulated datasets. The average results using the simulated datasets is compared to the results from the ground truth voter registry data.

| **Token A** | Voter Registry | Simulation | Difference |
| --- | --- | --- | --- |
| **FL** | 0.17 | 0.17 | 0.00 |
| **NC** | 0.17 | 0.15 | 0.02 |
| **OH** | 0.26 | 0.22 | 0.05 |
| **Tri-state** | 0.65 | 0.55 | 0.10 |
|  |  |  |  |
| **Token B** | Voter Registry | Simulation | Difference |
| **FL** | 0.19 | 0.19 | 0.01 |
| **NC** | 0.20 | 0.19 | 0.00 |
| **OH** | 0.39 | 0.37 | 0.02 |
| **Tri-state** | 1.12 | 1.12 | 0.00 |
|  |  |  |  |
| **Token E** | Voter Registry | Simulation | Difference |
| **FL** | 0.32 | 0.31 | 0.01 |
| **NC** | 0.29 | 0.27 | 0.02 |
| **OH** | 0.39 | 0.35 | 0.04 |
| **Tri-state** | 1.03 | 0.90 | 0.13 |
|  |  |  |  |
| **Token F** | Voter Registry | Simulation | Difference |
| **FL** | 0.66 | 0.64 | 0.02 |
| **NC** | 0.61 | 0.59 | 0.02 |
| **OH** | 0.83 | 0.81 | 0.03 |
| **Tri-state** | 2.72 | 2.62 | 0.10 |

Table 1: Re-identification rates in percentages for selected tokens based on actual voter registry data versus equivalent data generated by Monte Carlo simulation. The difference between the two is included for reference.

The differences consistently show a small reduction in re-identification risk from the simulated data compared to voter registry data. Therefore, the risk estimates from the Monte Carlo simulation should be considered a lower bound on the actual risk.
